# Production of Human Albumin in Pigs Through CRISPR/Cas9-Mediated Knockin of Human cDNA into Swine Albumin Locus in the Zygotes

**DOI:** 10.1038/srep16705

**Published:** 2015-11-12

**Authors:** Jin Peng, Yong Wang, Junyi Jiang, Xiaoyang Zhou, Lei Song, Lulu Wang, Chen Ding, Jun Qin, Liping Liu, Weihua Wang, Jianqiao Liu, Xingxu Huang, Hong Wei, Pumin Zhang

**Affiliations:** 1 State Key Laboratory of Proteomics, Beijing Proteome Research Center, Beijing Institute of Radiation Medicine, 27 Taiping Road, Beijing 100850, China; 2 Department of Laboratory Animal Science, College of Basic Medical Sciences, Third Military Medical University, Chongqing 400038, China; 3 Model Animal Research Center, Nanjing University, Nanjing, Jiangsu Province 210061, China; 4 National Center for Protein Sciences Beijing, Life Sciences Park, Beijing 102206, China; 5 Department of Science and Technology, Academy of Military Medical Sciences, 27 Taiping Road, Beijing 100850, China; 6 Center for Reproductive Medicine, The Third Affiliated Hospital of Guangzhou Medical University, Guangzhou, China

## Abstract

Precise genome modification in large domesticated animals is desirable under many circumstances. In the past it is only possible through lengthy and burdensome cloning procedures. Here we attempted to achieve that goal through the use of the newest genome-modifying tool CRISPR/Cas9. We set out to knockin human albumin cDNA into pig *Alb* locus for the production of recombinant human serum albumin (rHSA). HSA is a widely used human blood product and is in high demand. We show that homologous recombination can occur highly efficiently in swine zygotes. All 16 piglets born from the manipulated zygotes carry the expected knockin allele and we demonstrated the presence of human albumin in the blood of these piglets. Furthermore, the knockin allele was successfully transmitted through germline. This success in precision genomic engineering is expected to spur exploration of pigs and other large domesticated animals to be used as bioreactors for the production of biomedical products or creation of livestock strains with more desirable traits.

Human serum albumin (HSA) is the most abundant plasma protein that plays critical homeostatic functions in human physiology including maintenance of plasma oncotic pressure, regulating body fluids distribution, transportation of small molecules, etc^1^. It is prescribed for a number of severe diseases such as liver failure and traumatic shock^2^. Due to the shortage of human blood supply and the risks associated with human blood, alternative production of human albumin has long been sought. Recombinant HSA production was attempted previously in pigs through dominant transgene expression in the form of Albumin-GFP fusion^3^. However, in transgenic approaches, due to the presence of endogenous porcine albumin, separation and purification of the rHSA are problematic. Taking advantage of the power of CRISPR/Cas9 system in genome modification, we sought to produce rHSA in pigs through knocking human albumin cDNA into swine albumin locus. By inserting the human *ALB* cDNA plus SV40 polyA signal sequence (2368 bp total) into pig *Alb* locus immediately downstream the starting codon, we expect human *ALB* to be expressed under pig endogenous albumin transcriptional control and at the same time block the expression of pig endogenous albumin (Fig. 1a). We designed an sgRNA targeting the starting codon region (immediately 5′ of and including ATG) and generated a targeting fragment (donor for homologous recombination) with the insert flanked by 1 kb homology sequences on both sides (Fig. 1a). Since the 5′ homology sequence used in the donor runs up to the starting codon, it contains the sgRNA sequence and thus can be targeted by the sgRNA as donor or the knockin allele after homologous recombination. To prevent that from happening, we inserted 6 bp (gccacc) in the sgRNA sequence right before the starting codon. The sgRNA was transcribed *in vitro*, purified, and injected into the fertilized oocytes along with Cas9 mRNA^4^ and the circular vector containing the targeting fragment. The source of oocytes was Bama minipig^5^. The injected embryos were cultured for 1–2 hrs before being implanted in oestrus synchronized females. ~300 embryos were implanted in 10 females 5 of which became pregnant and delivered a total of 16 live pups. The pups were under standard care and did not show any signs of unusual health issues.

We clipped the ear tips of the 16 piglets when they were about 4-week old, and obtained genomic DNA for genotyping. To determine if we had succeeded in knockin, we assessed both 5′ and 3′ ends of the insertion site. We used two primer pairs, a/b and c/d (Fig. 1a). Primer a is outside the 5′-end of homology used and b is on human *ALB* (the insert); c is on human *ALB* and d outside the 3′-end of homology. As shown in Fig. 1b, all 16 piglets carry the intended knockin allele. We cloned and sequenced all of the DNA fragments amplified. They were the expected homologous recombination products (Fig. S1). Next, we determined the status of the wild-type allele in these piglets. Primers e and f (contained within the insert) (Fig. 1a) should amplify a 705 bp fragment from the wildtype locus and a 3085 bp fragment from the knockin allele. As expected, all of them generated the 3085 bp fragment. However, unexpectedly, we could obtain the apparent 705 bp wildtype fragment from only 7 (# 5, 6, 9, 10, 12, 13, and 15) of the 16 samples with the rest generating faint products around 700 bp (Fig. 1b). The apparent lack of wildtype allele in some piglets suggests that knockin might have happened on both alleles. Alternatively, the wildtype allele could have be edited in such a way that the primer a sequence was deleted, resulting in failure to amplify the wildtype allele. Indeed, editing on the wildtype allele did happen because the size of the amplified fragments differed from the predicted 705 bp in some piglets (Fig. 1b). To confirm that, we cloned and sequenced all amplified fragments. As shown in Fig. 1c, all of them were edited, although some were just a few base pair changes (hence appeared to be at 705 bp). Among these edited alleles, those found in piglet #10 and #12 are of interests. The one in #10 was the product of replacing a stretch of 29 bp (from the ATG towards 3′ end) of pig sequence with 36 bp (6 bp 5′ of the ATG and 26 bp afterwards) of the donor sequence. It is unclear how this happened. In #12, there are two different edited alleles, which puts total number of alleles at 3, indicating mosaicism in this piglet, which is not uncommon as mosaicism was often found in animals generated through zygote injection of Cas9/sgRNA^6^,^7^,^8^,^9^,^10^.

To determine whether off-targeting had occurred with the sgRNA, we chose 4 top potential off-target sites based on suggestions from CRISPR design tool (http://tools.genome-engineering.org) and amplified fragments containing these sites from the ear tip genomic DNA of piglet #14. The fragments were subjected to T4EN I assay^4^. As shown in Fig. S2, no off-target editing was found. However, we could not eliminate the possibility that off-target editing happened in other loci or in the other 15 transgenic pigs. Nonetheless, even there were off-target editing, the edited loci are unlikely to be problematic for our purpose of producing rHSA, as long as they do not interfere with the welfare of these transgenic pigs.

Having demonstrated successful knockin of the human *ALB*, we sought to determine if human albumin could be detected in the blood plasma of these knockin piglets. As shown in Fig. 2a, all of the piglets contained human albumin in their blood plasma detectable with the antibodies specific against human albumin, albeit at variable levels, which is likely a result of at least two factors, whether both alleles are knockin and the extent of mosaicism (especially in the liver). The level in #7 is very low only visible after long exposure of the blot, despite the apparent lack of wild-type pig *Alb* allele (Fig. 1a). Overall, the levels are much lower than that in adult human sera. It is known that the concentration of blood albumin in pigs increases with age, reaching a level similar to that in adult humans by 6 months of age^11^.

To confirm that the rHSA detected in the plasma of our knockin piglets with antibodies is truly human albumin, we subjected two plasma samples (piglet #2 and #6) to mass spectral analysis. #2 is apparently homozygous for the knockin allele and #6 contains one knockin and one mutant allele (frameshift) (Fig. 1). 0.5 μl plasma was separated on SDS-PAGE, and proteins around 70 KD were in-gel digested with trypsin. The tryptic peptides were eluted out of the gel, dried and re-dissolved for separation by liquid chromatography and mass spec analysis. For piglet #2, we could detect 14 unique human ALB tryptic peptides and 15 for #6 (Fig. 2b). M/Z spectra for human peptide FKDLGEENFK and pig peptide FKDLGEQYFK (both from piglet #2) were shown in Fig. S3. Two peptides were chosen for quantification by measuring the peak areas. In agreement with the western blot results, these two peptides were much more (~5 times) abundant in #2 than in #6 (Fig. 2c). These results demonstrate that the rHSA detected by antibodies is authentic human albumin.

Genotyping of the DNA isolated from ear tips indicates that both #2 and #6 contain no wildtype pig *Alb* allele, either not present or edited (Fig. 1b,c). However, we could still detect tryptic peptides from pig albumin in both samples, but at much lower abundance (Fig. 2c). Interestingly, the abundance of pig peptides was about the same between the two samples, despite that the abundance of human peptides differed greatly. These results suggest that both piglets contain a similar number of wildtype (or heterozygous) hepatocytes which are responsible for the pig ALB detected in the blood of these two piglets. However, such wildtype allele-containing cells might not exist or exist in an extremely low percentage in the ear tips so that the wildtype allele could not be PCR amplified. Further, Southern blot analysis with an internal probe (3′ half of human *ALB* CDS) indicated the presence of an additional insertion of the donor sequence in piglets #1, 4, and 5 (Fig. S4). All these complications will not be an issue for the purpose of producing recombinant human albumin as the knockin allele can be purified through backcrossing.

To determine if the knockin allele can be transmitted to next generation, we crossed #2 (male, homozygous) with #5 (female, heterozygous) when they became reproductively mature. 6 pups were born out of the cross. 4 of them (#1, 3, 4, and 6) are homozygous for the knockin allele (*Alb*^*H/H*^, H denotes human) and 2 (#2 and 5) heterozygous (*Alb*^*P/H*^, P denotes pig) (Fig. 3a). #5 and 6 died of diarrhea about 2 weeks after birth. We analyzed human albumin expression in the blood of the remaining piglets at 4-week of age. As shown in Fig. 3b, all of them have human albumin in their blood. Interestingly, the level of human albumin in the heterozygous animal (#2) was much lower than that in the homozygotes. We do not know if this is a result of individual variation or the knockin allele is somehow suppressed by the wildtype allele.

We show here the successful generation of pigs carrying human *ALB* cDNA knocked into porcine *Alb* locus. This is one step further than the simple gene editing in pig zygotes reported recently by Hai *et al.*^12^ and us^13^. Large domesticated animals have been pursued as bioreactors for biomedical protein products^14^,^15^,^16^,^17^,^18^,^19^,^20^. Usually, the coding sequences of these proteins were inserted randomly along with necessary transcription control elements into the genome as transgenes, which is associated with many complications including the short-term nature of transgene expression. Our demonstration that homologous recombination can occur highly efficiently in pig zygotes opens the door for the development of ever better bioreactors as well as livestock strains with more and more desirable traits.

## Methods

### DNA Constructs

The 5′ and 3′ homology arms were PCR-amplified with KOD FX DNA polymerase (TOYOBO, KFX-101) from pig genomic DNA. Human albumin CDS was obtained from IMAGE collection. The homology arms, human *ALB* CDS, and SV40 polyA signal sequence were assembled together to generate the donor plasmid. The sgRNA (AAGCCTTTGGCACAATGAAG) was synthesized as an oligo linker and cloned into the pUC57-sgRNA expression vector^4^. The production and purification of Cas9 mRNA and sgRNA were performed as described^4^.

### Animals

The pigs used in this study were maintained in the Laboratory Animal Centre of the Third Military Medical University. All of the protocols involving the use of animals were approved by the Institutional Animal Care and Use Committee of the Third Military Medical University (Approval ID: SYXK-PLA-2007036). The experiments were carried out in accordance with the approved protocols and guidelines.

### Microinjection of pig zygotes

Pig zygotes at one- or two-cell stage were surgically collected from mated sows as described^5^,^13^,^21^. The collected zygotes were subjected to cytoplasmic microinjection with the mixture containing Cas9/sgRNA/targeting plasmids at 20, 10, and 35 ng/μL, respectively. A total of 300 zygotes (193 at 1-cell and 107 at 2-cell stage) were injected. For zygotes at 2-cell stage, both cells were injected. Shortly after the injection, the zygotes were transferred into synchronized foster female sows as described^5^,^13^,^21^. Pregnancy was monitored by observing oestrus behavior of the recipient sows at every ovation circle.

### PCR Genotyping and T7EN1 Cleavage Assay

**Figure 1:**
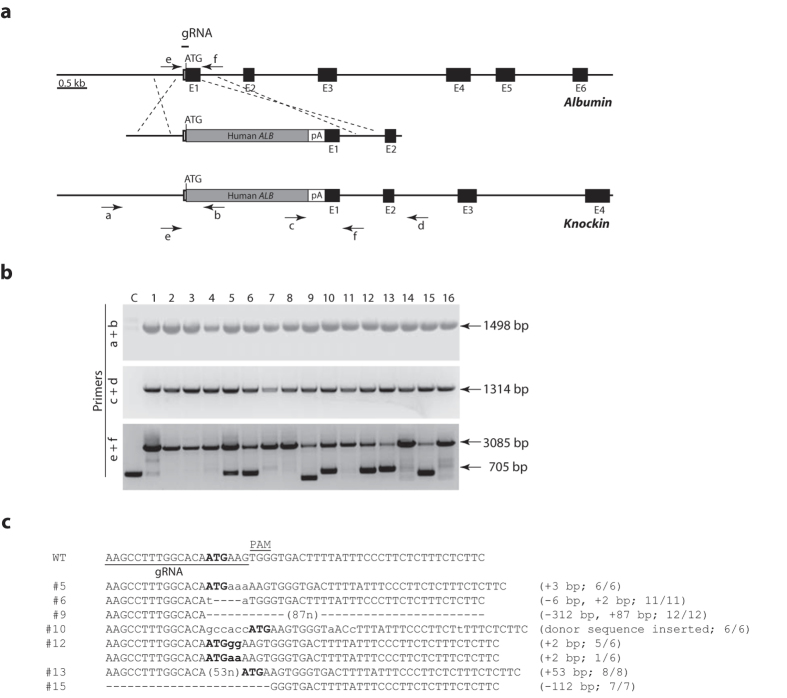
Knockin of human *ALB* into porcine *Alb* locus. (**a**) Diagram of the knockin strategy. (**b**) Genotyping of the founders. PRC primers are illustrated in (**a**). (**c**) Sequencing results of the PCR products obtained with primer pair e/f (**a**). The products were cloned and up to 12 clones (for #9) were sequenced.

The genomic DNA was extracted from tissue lysates by phenol-chloroform and recovered by alcohol precipitation. PCR primers used (Fig. 1a) are: a, 5′GCTGTGGAAACGCCTTAACC3′; b, 5′AGCAGTCAGCCATTTCACCA3′; c, 5′TCTCTTATTCCACTTCGGTA3′; d, 5′ATTTAAAGTACTCCGTAGCC 3′; e, 5′ ACAGATCCAGACGGCAAACA 3′; f, 5′AGCTACTGAGAGGATGGTCTG3′.

T7EN1 cleavage assay was performed as described^4^. In brief, targeted fragments were amplified with KOD FX DNA polymerase from extracted genomic DNA, and purified with PCR cleanup kit (OMEGA, D2500-01). The purified PCR products were denatured and re-annealed in NEBuffer 2 (NEB), and digested with T7EN1 (NEB, M0302L) for 30 min and separated by 2% agarose gel.

### Southern Blot Analysis

For Southern blot analysis, the ear tip genomic DNA was digested with *Nde* I to completion, separated on 0.8% agarose gel, blotted to charged nylon membrane under denaturing conditions. To label the probe, we performed a PCR reaction with DIG Probe Synthesis Kit (Roche). The primers were SNB030-F, 5′GCTATGCCAAAGTGTTCG3′ and SNB030-R, 5′AAGCAGGTCTCCTTATCGT3′ and the template was the donor plasmid. The probe is 592 bp long. The blots were hybridized with the probe and the labeled bands were detected with the digoxin hybridization detection kit (Roche).

### Immunoblotting and Mass Spec Analysis

Plasma samples were mixed with 5x SDS loading buffer, boiled for 2 min, separated by SDS-PAGE, and transferred to nitrocellulose membrane (Millipore). The blots were incubated with antibodies for albumin (CST, # 4929S), washed, and incubated with a horseradish peroxidase-conjugated secondary antibody. The secondary antibody was detected with enhanced chemiluminescent (ECL) substrates (Pierce). The blots were visualized with ImageQuant LAS500 (GE).

Mass spec analysis was performed according to Ding *et al.*^22^. High confidence (corresponding to≤1% false discovery rate for peptide spectrum matches), minimum peptides length of 7 amino acid residues, peptide score 10, and peptide rank 1 were used to filter the result matches.

## Additional Information

**How to cite this article**: Peng, J. *et al.* Production of Human Albumin in Pigs Through CRISPR/Cas9-Mediated Knockin of Human cDNA into Swine Albumin Locus in the Zygotes. *Sci. Rep.* **5**, 16705; doi: 10.1038/srep16705 (2015).

## Supplementary Material

Supplementary Figures

## Figures and Tables

**Figure 2:**
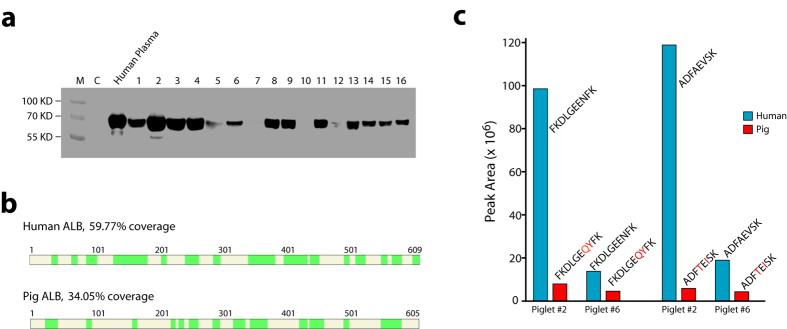
Analysis of human ALB in the blood. (**a**) Western blot detection of human albumin in the blood of founder piglets. 0.5 μl plasma from each founder was separated on SDS-PAGE and analyzed. Human blood plasma (0.5 μl after diluted 30 times) was used as a positive control. C, plasma from a wildtype pig. (**b**) Illustration of tryptic peptides (green) detected with mass spec analysis. (**c**) Semi-quantitation of two tryptic peptides from human and pig albumin through measuring the peak areas of each peptide. Red marks the amino acid residues that are specific to pig.

**Figure 3:**
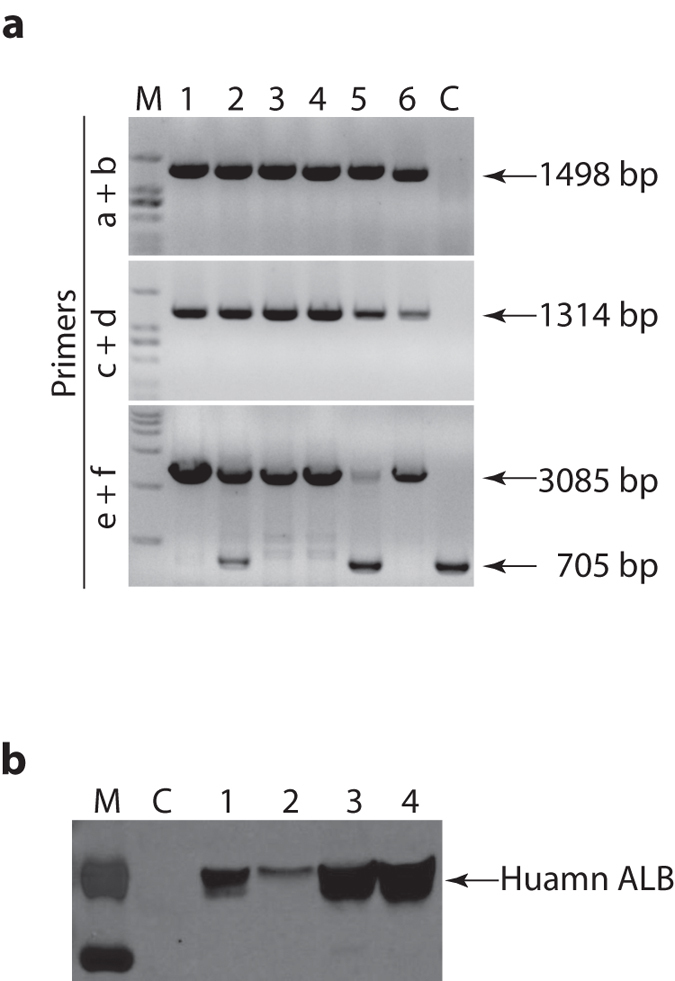
Germline transmission of the knockin allele. (**a**) PCR genotyping of the F1 offspring. Primers used were the same as in Fig. 1. (**b**) Western blot detection of human albumin in the blood of the F1 piglets. 0.5 μl plasma from each piglet was separated on SDS-PAGE and analyzed. C, plasma from a wildtype pig. M, molecular weight marker.
