# Supplementary material for: Production of Human Albumin in Pigs Through CRISPR/Cas9-Mediated Knockin of Human cDNA into Swine Albumin Locus in the Zygotes
Source: Sci Rep. 2015 Nov 12;5:16705. doi: 10.1038/srep16705 (PMC4642324; doi:10.1038/srep16705)
Supplement: Supplementary Figures [file srep16705-s1.pdf]

Production of Human Albumin in Pigs Through CRISPR/Cas9-Mediated Knockin of Human cDNA into Swine Albumin Locus in the Zygotes

Jin Peng[1#], Yong Wang[2#], Junyi Jiang[1], Xiaoyang Zhou[2], Lei Song[1], Lulu Wang[2], Chen Ding[1], Jun Qin[1], Liping Liu[5], Weihua Wang[6], Jianqiao Liu[6], Xingxu Huang[3], Hong Wei[2 *], and Pumin Zhang[1, 4 *]

Supplemental Figures

a

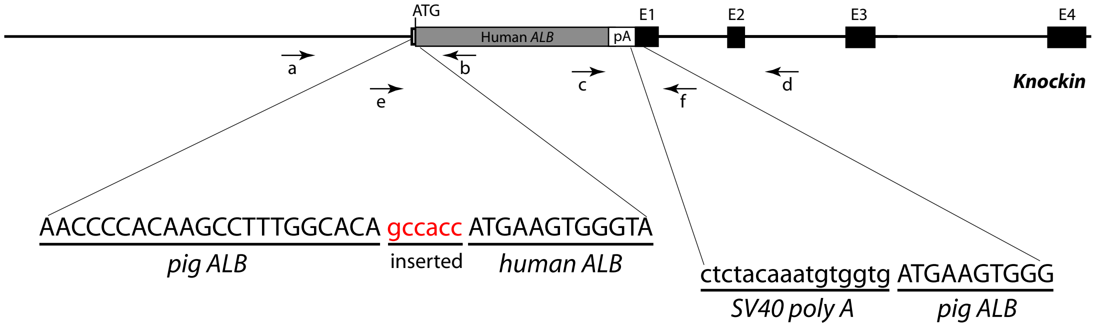

Wildtype pig *ALB*

—AACCCCACAAGCCTTTGGCACA-----------ATGAAGTGGGTG

gRNA

b

a+b, 1498 bp (green, 5′ homology arm; red, inserted sequence; blue, human *ALB*)

```
gctgtggaaacgccttaacccattgcaccaccagggagcttcatgaaatccatagttcttcatctttagagcaaaagggcagagagagaatagtc
cttcagaggcagagatcagaatttggggtgggaaaagtaagGAAGGAAGTTCGAAAGAACATTTTTGAAAAATTATCTTTGTTTTTAGACATGAA
GTTAAATTTATTTCGAAATTTAAAATAACATTTAAGAAGAGCATGTGACTTTCCTTTTTAGTAAAAGCGAGAAAGAAAGAAAAATGTGAAATAGG
GATGGAAAGAATATAATGCCTGGTAAAGAAGGTCTAGGATTCTTAAAACCACTAATATAATCAGGCTCAGCCTGCCCTGTGTATATCATGATAGA
AAAGATTACCACTGCTGCCATTGTGTCCATTTTCTTCCCCATATCTCCATGAGAGGTACCATACTGCCTCTAAGTACTTTACTCTAGAGCCTGAA
TCACCAGTGGGCCAGAGCTGAGGTAAAAATGTAGGACTTCTCAACATCCGTTTTAGTTGTTATTGTTGCTATGACTTTTTCTTAGTTTGCATTTG
AGACACTGGTTTTTTTGACAGAACAGTTTCAGAAGAATGAAAAGCCATGGAAGCCCTCGAATGCGTGCATGCATGCATGCATGCATGCACACACC
CATGTGTGTATGCATTTTCACTACTTATCAATAAAAGCATACTTGGAATTAATAATAGATACAATTCAGAGCTTAGCTCTTTTAACATGTCTTAT
TTCCAAGCTAAGTCACTGGTTTGCTTAAATTTTTGCAAATTTTGAAAACTAACACGTTCAAATGGTAAACCACAGATAGAGATCATGGTAATAGT
ACAAATCGTTTAAACTCTTATGTAAAATTTGATAAGGTGTTTTACATAACTTTAATGAATTGACAAGGTCTTGTGGAGAAAACAGATCCAGACGG
CAAACATACGCAAGGGATTTAGTCAAACACATTTTTGGCAAAAAAACTATGAATTTTGTAATCAGTTGTGAGCCAATGAAATACAAAAATGAGTC
TAGTTAATAATCTACAATTATTGGTTAAAGAAGTATATTAGTGCTGACTTTCCTCTGTTCGTCCTACCTTTTCTCTTCTATCAACCCCACAAGCC
TTTGGCACAgccaccATGAAGTGGGTAACCTTTATTTCCCTTCTTTTTCTCTTTAGCTCGGCTTATTCCAGGGGTGTGTTTCGTCGAGATGCACA
CAAGAGTGAGGTTGCTCATCGGTTTAAAGATTTGGGAGAAGAAAATTTCAAAGCCTTGGTGTTGATTGCCTTTGCTCAGTATCTTCAGCAGTGTC
CATTTGAAGATCATGTAAAATTAGTGAATGAAGTAACTGAATTTGCAAAAACATGTGTTGCTGATGAGTCAGCTGAAAATTGTGACAAATCACTT
CATACCCTTTTTGGAGACAAATTATGCACAGTTGCAACTCTTCGTGAAACCTATGGTGAAATGGCTGACTGCT
```

c

c+d, 1314 bp (blue, human *ALB;* red, SV40 poly A; green, 3′ homology arm;)

```
TCTCTTATTCCACTTCGGTAGAGGATTTCTAGTTTCTTGTGGCTActcgagCAGACATGATAAGATACATTGATGAGTTTGGACAAACCACAAC
TAGAATGCAGTGAAAAAAATGCTTTATTTGTGAAATTTGTGATGCTATTGCTTTATTTGTAACCATTATAAGCTGCAATAAACAAGTTAACAACA
ACAATTGCATTCATTTTATGTTTCAGGTTCAGGGGGAGGTGTGGGAGGTTTTTTAAAGCAAGTAAAACCTCTACAAATGTGGTGATGAAGTGGGT
GACTTTTATTTCCCTTCTCTTTCTCTTCAGCTCTGCTTATTCCAGGGGTGTGTTTCGTCGAGATACATGTAAGAGTTCGATTTTTCTATTGTTCA
ACTTTTATTTTCCTAGTAAAAGAAAGTTTCAGTAAACTTTGAATCTTTGATGAATTAAGTTTTCCTTTAGCATTTATTTCTAGACTGTTATGATA
TTTTGTATTTGCGAACCCTTAGGAAGTTATCTTATTAATATGTTTCTAACATGCTGGGTGAAAAAATAGTGAGAATTGTTTAGTAGCTATAATTT
TCTTTTGCACATGAAGGAGCTTATGTAGAGTGACTTACACTTCACAGAGTGCAGTTGAAGACAGAATTAAAAATTCCAAATTCCTAAAGGCCTAT
TCACTGTACCTTACACCTTGGTTTATATTAAGAATATACAAACACTTTATAAAAAGAATTTGCATTTATATTTTTCATTTCAGACCATCCTCTCA
GTAGCTGGTAATAGATACTAAATGAACATTAGATCTTATCCAAGTTTGAATAGAAAGCCATAAATATTTAATGATTTTAATGGTTTTTCAAATAA
TATCATGGGCAATTAAATAACTCTTCTGTTTAAATGCAAAAGAAGAAATTTACCACCGTCCTAAGATTTTCTGTAAAGATCAGAGCCCAGTATCT
AGGAGCAAATGAATACCTAAGTCAAATGAAGAGAAACAAAAAATATTATCTTAACTGGTTGTTTCGTCAGAATTTAAGCAAAACTTTTTTTCTCT
CCTCTTTTCCCCAGACAAGAGTGAAATTGCTCATCGGTTTAAAGATTTGGGAGAACAATATTTCAAAGGCCTGTAAGTTAAAATATTGAAGATTC
AAATTTAATGTTTCTATTAGTGTCAGTTATTATTTTGACACCCTCATATTCCCCCGCCATCAGAGATTCTAGAGCAGTCCCTCCAGTAGAATTTT
CTGCGTTGGTGGAAATATCCTCTATTGGTGTTGTCTGACagttgccattggccacatgtggctacggagtactttaaat
```

Figure S2

a

```
        sgRNA  AAGCCTTTGGCACAATGAAGTGG

Off-target 1   AAGCCATTTGCACAATGAAGAAG
Off-target 2   AGACCTTTGGCACAATGAATGAG
Off-target 3   CAGCCTTAGGCACAATTAAGTAG
Off-target 4   AAGCATGTAGCACAATGAAGAAG
```

b

```
OFF 1F   GGGAATAAAATGCTTAGAACTGGCT
OFF 1R   TTCCAACTGGCTCAAGACCAT

OFF 2F  GACTATCTGAGGTGTGCAGAGG
OFF 2R  CTCAATACATCAGGGCACGC

OFF 3F  AAGGCTACCATTACGGTGTGA
OFF 3R  TCGGGGGAGCTTCTGACTTT

OFF 4F  CAGGGGAGCACTACCTAGACT
OFF 4R  AGGCAGTCCTGAGATCGGAAA
```

c

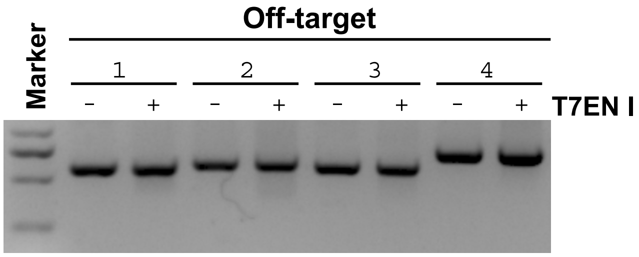

Figure S3

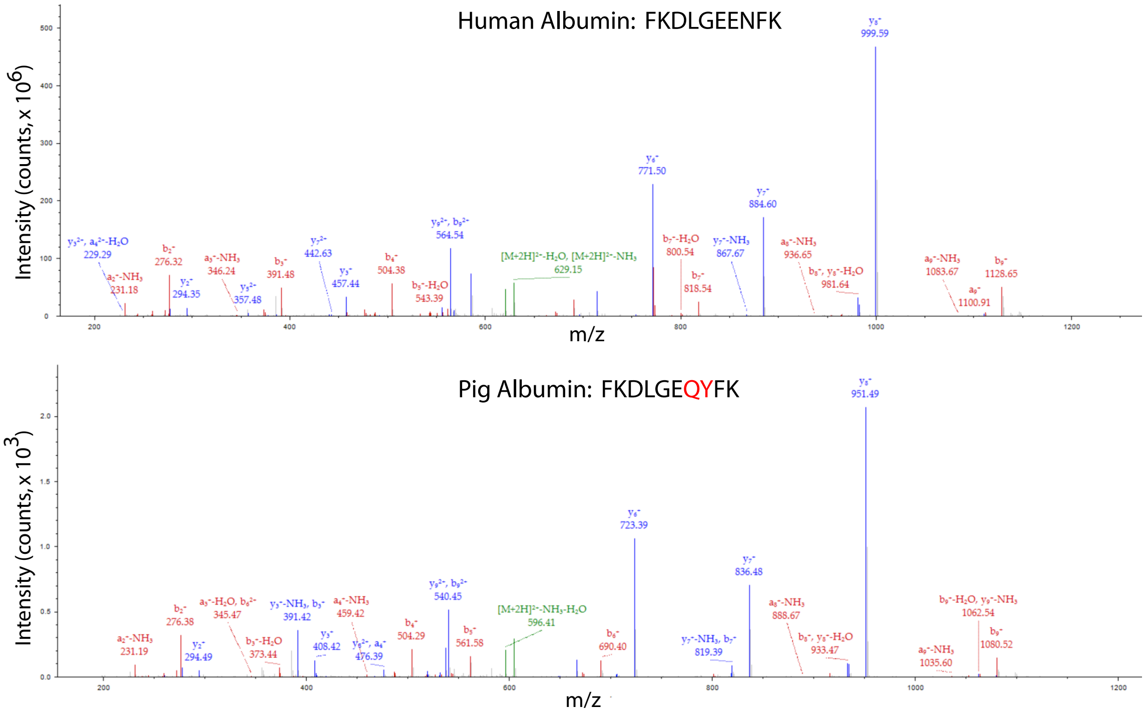
Human Albumin: FKDLGEENFK
Intensity (counts, x 10^6)
m/z
Pig Albumin: FKDLGEQYFK
Intensity (counts, x 10^3)
m/z

Figure S4

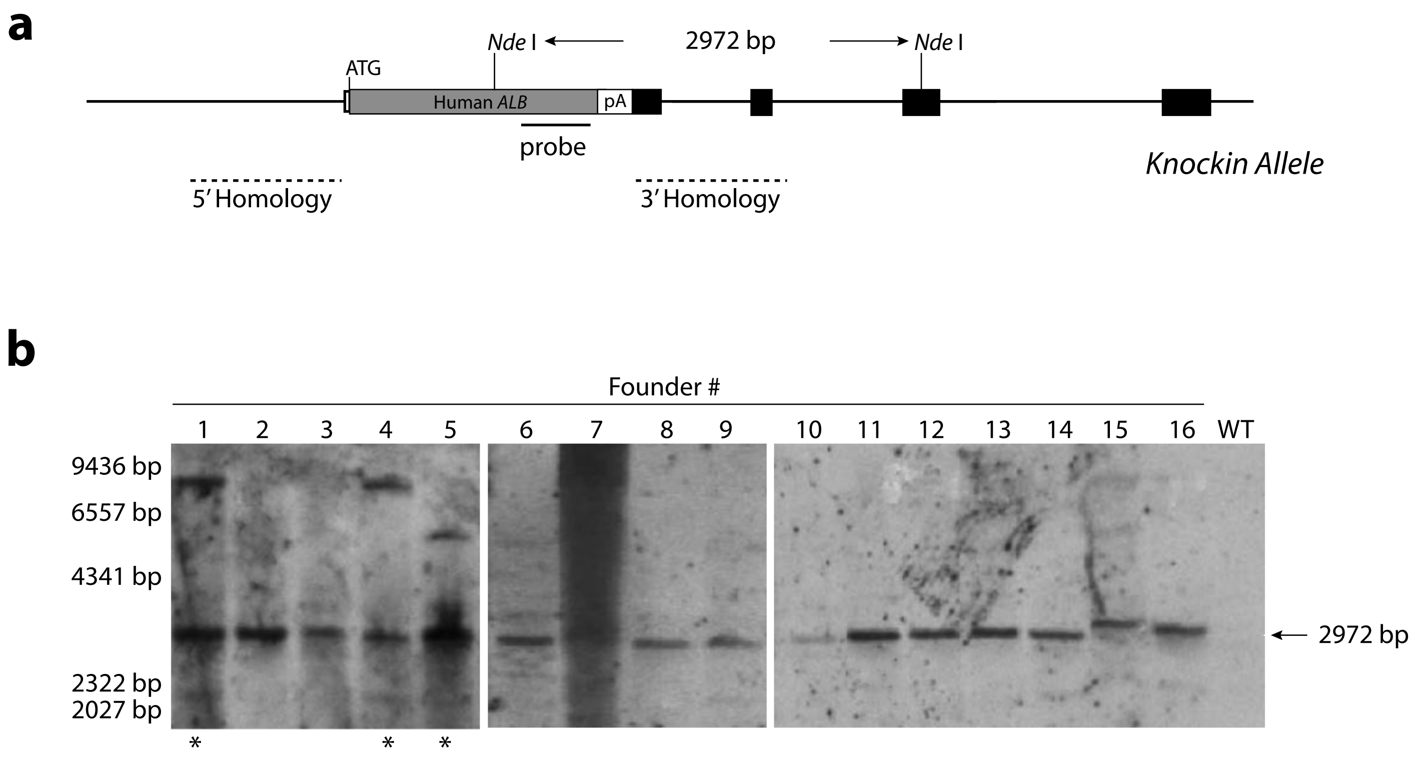
a
Nde I
2972 bp
Nde I
ATG
Human ALB
pA
probe
5′ Homology
3′ Homology
Knockin Allele
b
Founder #
1 2 3 4 5 6 7 8 9 10 11 12 13 14 15 16 WT
9436 bp
6557 bp
4341 bp
2322 bp
2027 bp
2972 bp
* * *

Figure S1. Sequencing analysis of homologous recombination. a. Illustration of the knockin allele. Sequences of the 5' and 3' junctions of the transgene were shown as well as the relevant pig *ALB* sequence. **b.** Sequence of the PRC product covering the 5' junction between pig *ALB* and the 5' homology arm. **c.** Sequence of the PCR product covering the 3' junction between the 3' homology arm and pig *ALB*.

Figure S2. Off-target analysis. a. Sequences of the sgRNA and 4 top potential off-target sites. **b.** Primer sequences used to amplify genomic fragments containing the 4 potential off-targets. **c.** T7EN I assay of the PCR fragments containing the 4 sites.

Figure S3. Mass spectra of human albumin peptide FKDLGEENFK and pig albumin peptide FKDLGEQYFK.

Figure S4. Southern blot analysis. a. Diagram of the knockin allele. **b.** Analysis of founder genomic DNA. The genomic DNA was completely digested with *Nde* I. Piglets #1, 4, and 5 (indicated by *) contain additional insertion of the human *ALB* sequence. The *Nde* I fragment in piglet #15 seemed larger than the predicted 2932 bp the reason for which is unclear at present. The genomic DNA from piglet #7 was degraded.
